# Characterizing modifications to the mental health gap action programme (mhGAP) intervention guide during implementation in low- and middle-income countries using the framework for reporting adaptations and modifications to evidence-based interventions: a systematic review of reviews

**DOI:** 10.1186/s13033-025-00671-z

**Published:** 2025-07-02

**Authors:** Harikeerthan Raghuram, Akanksha Jayant Rajguru, Mythili Menon Pathiyil, Aakrushi Brahmbhatt, Anant Bhan, Jessica Spagnolo, John A. Naslund

**Affiliations:** 1 Sangath, Bhopal, India; 2 AIIMS, New Delhi, India; 3 Université TÉLUQ, Québec, Canada; 4 Centre de recherche Charles-Le Moyne, Longueuil, Québec Canada; 5 Department of Global Health and Social Medicine, Harvard Medical School, Boston, USA

**Keywords:** MhGAP, Systematic review of reviews, Implementation, Global mental health, Modification, LMICs, Task-sharing

## Abstract

**Background:**

Low- and middle-income countries (LMICs) allocate a disproportionately small fraction of their healthcare budgets to mental health, leading to a treatment gap exceeding 75%. To address this disparity, the World Health Organization (WHO) introduced the Mental Health Gap Action Programme (mhGAP), aiming to integrate mental healthcare into primary and community care settings. Central to this initiative is task-sharing: empowering non-specialist healthcare providers to detect and treat mental disorders. Adaptation and modification of mhGAP to the national and local contexts is an integral aspect of the guidelines.

**Methods:**

This systematic review of reviews employs the Framework for Reporting Adaptations and Modifications-Expanded (FRAME) to document and characterize modifications to mhGAP implementation in LMICs. The databases searched included Embase, PubMed, PsycINFO, CINAHL, Google Scholar, Cochrane, and Web of Science. Reviews selected in stage 1 were used to find empirical studies from which relevant data was extracted.

**Results:**

Narrative synthesis suggests that modifications primarily focus on content, delivery, and training methods, with limited attention to scaling up. Modifications adopt top down, yet consultative and participatory approaches. There is a notable lack of reporting on challenges, processes, and outcomes. Recommendations have been made to expand FRAME, namely, sources of knowledge, financial and temporal resources employed during the process of modification.

**Conclusion:**

Modifications are essential for adapting interventions to diverse settings, yet they are often researcher-led with limited stakeholder involvement. Better documentation—particularly on challenges and outcomes—is needed. Strengthening frameworks like FRAME can improve reporting, optimize resources, and enhance implementation and scale-up in similar contexts.

## Introduction

Approximately 14% of the global disease burden is attributed to Mental, Neurological, and Substance Use disorders (MNS) disorders, with a disproportionate share in LMICs. Despite this burden, many low-and middle-income countries (LMICs) allocate less than 2% of their total healthcare budgets to mental health, contributing to a treatment gap exceeding 75% [1]. This is largely related to a shortage of trained mental health professionals [2, 3]. In response, the World Health Organization (WHO) introduced the Mental Health Gap Action Programme (mhGAP) in 2008 [4]. This initiative aims to bridge the treatment gap by facilitating the integration of mental health care into primary care and community-based settings, primarily achieved by training existing non-specialist health care providers in the detection of mental disorders and the delivery of evidence-based treatment options [5, 6]. mhGAP encompasses a comprehensive set of guidelines and tools designed for prevention and management of MNS disorders, enhancement of availability and accessibility of mental health services by engaging national governments, international organizations, civil society, and the community [4]. Since the launch in 2008, WHO further released a linked Intervention Guide in 2010, a version 2.0 in 2016, and an operations manual in 2018 as tools to further support capacity-building in primary and community-based settings [1, 4, 7].

The mhGAP and its accompanying guide (mhGAP-IG) are meant as a general guide that is to be modified to meet the specific requirements of both national and local contexts [1, 4]. Therefore, one crucial aspect for implementation science is the modification of interventions to suit local contexts and needs. Modifications help align interventions with the needs of the target population in a specific system or context which may in turn lead to improved engagement, better reach, higher acceptability, and enhanced outcomes [8]. They can be planned and proactive; or unplanned and reactive [9]. Planned and proactive modifications are generally referred to as adaptations.

Despite the large number of studies that have documented the mhGAP implementation in LMIC settings, there are surprisingly few studies that have characterised the modifications to mhGAP [10]. For example, a 2017 review uncovered 33 peer-reviewed articles on mhGAP implementation, only three of which described modifications [10]. This review was updated in 2021 and found 162 new articles of which only 13 described modifications [11]. This is in line with other studies which suggests inadequate reporting and documentation of adaptations [12, 13]. Insufficient reporting of modifications not only hampers the advancement of the science of modifications but also impedes the further refinement of mhGAP. Comprehensive reporting and documentation of modifications hold great significance as they can provide vital information explaining the heterogeneity in factors affecting implementation outcomes across sites. This information can then be used to optimize implementation outcomes, reduce scaling-up expenses, enhance intervention delivery by streamlining the evaluation and modification process, minimize the costs of adapting and implementing interventions in culturally similar contexts, bolster replicability and mitigate or prevent duplication of efforts [14, 15].

This systematic review, therefore, leverages the Framework for Reporting Adaptations and Modifications-Expanded (FRAME) [16] to identify and characterize modifications to mhGAP and mhGAP-IG implementation in LMICs. FRAME, proposed by Stirman and colleagues, is an updated version of their original framework [16] and was conceived with the specific purpose of providing systematic guidance for tracking and characterising adaptations and modifications during implementation processes [17]. The framework has facilitated the methodical documentation of the processes and the monitoring of modifications applied to evidence-based practices, particularly within healthcare contexts. Its utility has been demonstrated in various interventions, such as Montessori-based activity programming [18], Skills Training in Affective and Interpersonal Regulation (STAIR) for posttraumatic stress disorder (PTSD) for peer delivery [19], lung cancer screening within the Veterans Health Administration [20] and Savvy Caregiver Program (SCP) for Korean American dementia caregivers [21], among others. The FRAME has also been used as the basis for enquiry for key informant interviews aimed at improving uptake, scalability and sustainability of interventions [22].

This review aims to comprehensively document modifications to mhGAP using FRAME, understand the nature and processes involved in modifications, and potentially identify domains for documenting modifications in addition to what is there in FRAME.

## Methods

The systematic review protocol is registered in the International Prospective Register of Systematic Reviews (PROSPERO 2021 CRD42021284089) and follows the Preferred Reporting Items for Systematic Reviews and Meta- Analyses (PRISMA) guidelines [23].

### Review approach

This review can be characterised as a systematic review of reviews as it reviews existing reviews. We use the term *modification* as used by Stirman and colleagues [24] where it is used as an overarching term including planned modifications with an intention for better fit (adaptations) and unplanned modifications. Data synthesis for this review adhered to the *best fit framework synthesis* approach proposed by Booth and Carrol [25]. This approach requires the identification of an existing framework for synthesis and FRAME [16] was chosen since it examines and records the types of modifications, processes involved and reasons that necessitated them. A meta-analysis was deemed inappropriate owing to the variability of studies with respect to the heterogeneous study populations, aim and objectives, research designs, and outcomes.

### Search strategy

We searched Embase, PubMed, PsycINFO, CINAHL, Google Scholar, Cochrane, and Web of Science from 01/01/2008 until 31/08/2021, with the following search terms: ‘mental health gap action programme’ OR ‘mental health gap action program’ OR ‘mhGAP.’ 2008 was selected as start date, given that it was the launch year for the mhGAP [1]. These databases were selected by the authors based on databases that the authors had access to and with an attempt to be as exhaustive as possible.

### Study selection

All identified reviews were included in a Microsoft Excel file, which was used to remove duplicate reviews and to conduct review screening. Three reviewers (MP, AB, and HR) reviewed titles and abstracts independently, followed by independent full-text review of shortlisted reviews. Disagreements were resolved through discussion until consensus was reached along with discussions with JN and JS when required. During full-text review in the first stage, we excluded reviews that did not mention mhGAP, were not implemented in LMIC, were protocols, were non-reviews, and had primary data collection as a study objective. Studies in any language within these databases were included. The classification of a country as LMIC was determined based on the 2018 World Bank classification criteria [26]. We defined reviews as any empirical study using published studies as the source of data without primary data collection. Hence, some commentaries were included as they fit our broad criteria of narrative reviews which we defined as any peer reviewed study that includes a literature review but does not include primary data collection.

In a second stage, MP then extracted all the studies included in the reviews that met eligibility criteria. From the included systematic reviews, we extracted only those studies that were reviewed in the systematic review and not all the studies in the reference list. For commentaries we selected all the studies in the reference list. A second screening was done to remove study duplicates retrieved from the eligible systematic reviews. In addition, we removed those that were not empirical studies, did not take place in LMIC, were commentaries, reviews, and protocols. Thus, while in the first stage screening we included only reviews and excluded studies with primary data collection, in the second stage screening from within the reviews we excluded reviews and included only empirical studies with primary data collection. We allowed for a broad definition of empirical studies where we included any studies involving primary data collection regardless of the methods used: observational studies, qualitative studies, quasi-experimental studies, randomised controlled studies among others.

Subsequently, we used the following criteria to select studies that mentions adaptations/ modifications to mhGAP. For this, the following terms were searched within the full text of the articles: modification(s), adaptation(s), contextualisation, tailoring, alteration, adjustment, change, improvement, refinement, revision, refashioning, transforming, customisation, revamping, remodelling, reshaping, integration, fitting, tuning, and tweaking. These terms were chosen based on a preliminary reading of modification literature and using dictionary synonyms of the term modification. Studies that mentioned any of the above terms and had sufficient information on modification were then selected for data extraction.

### Data extraction

A full text review was implemented to assess the eligibility of the studies based on our inclusion and exclusion criteria, post which data were extracted using an extraction table in Microsoft Excel. The following variables were first extracted to capture information: (i) Author; (ii) Year; (iii) Type of Study (Qualitative/ Quantitative/ Mixed Methods); (iv) Summary of the findings; (v) Country; (vi) Region; (vii) Study Settings (primary/ secondary/ tertiary centres/ community).Subsequently, additional variables were extracted on the basis of the eight elements described in the FRAME [16] (See Table 1 below).

**Table 1:** FRAME elements in the data extraction table

FRAME element	Definition
(i) When and how in the implementation process the modification was made (Timing of modification)	Focusing on the timing of making modifications during implementation
(ii) Whether the modification was planned/proactive or unplanned/reactive	Distinguishing between planned and unplanned modifications can provide insights into the intention behind the changes. Planned modifications are made deliberately, while unplanned ones may be in response to unforeseen circumstances.
(iii) Who determined the modification?	Identifying who made the decision to modify the intervention.
(iv) What is modified?	Specifying the elements of the intervention that were modified or adapted. It could include changes to intervention content, delivery methods, or other components.
(v) At what level of delivery the modification is made	Understanding where the modification occurs in the delivery process (e.g., at the provider, organization, or system level)
(vi) Type or nature of content-level modifications	Better understanding the specific type or character of changes or adaptations made to an intervention or program during its implementation.
(vii) Relationship to fidelity	Assessing the fidelity-consistency of modifications can help to determine whether the changes maintain fidelity to the original intervention or diverge from it.
(viii) Goal/ rationale for the modifications made	Including both the intent or goal of the modification (e.g., improving fit, cultural adaptation, cost reduction)

In addition to the domains in FRAME, the authors also extracted data pertaining to steps taken to formulate the modification of the intervention. This included the processes followed during the course of preparing for, developing modifications to interventions, additional sources employed to develop modifications. All articles that met eligibility criteria were read in full and data were extracted by one reviewer (AR), with a second reviewer independently reviewing and validating all data extractions (HR). Any discrepancies between the interpretations of different reviewers were discussed in regular group meetings until a consensus was reached. Doubts were cleared in team meetings with JN and JS.

## Results

**Fig. 1:**
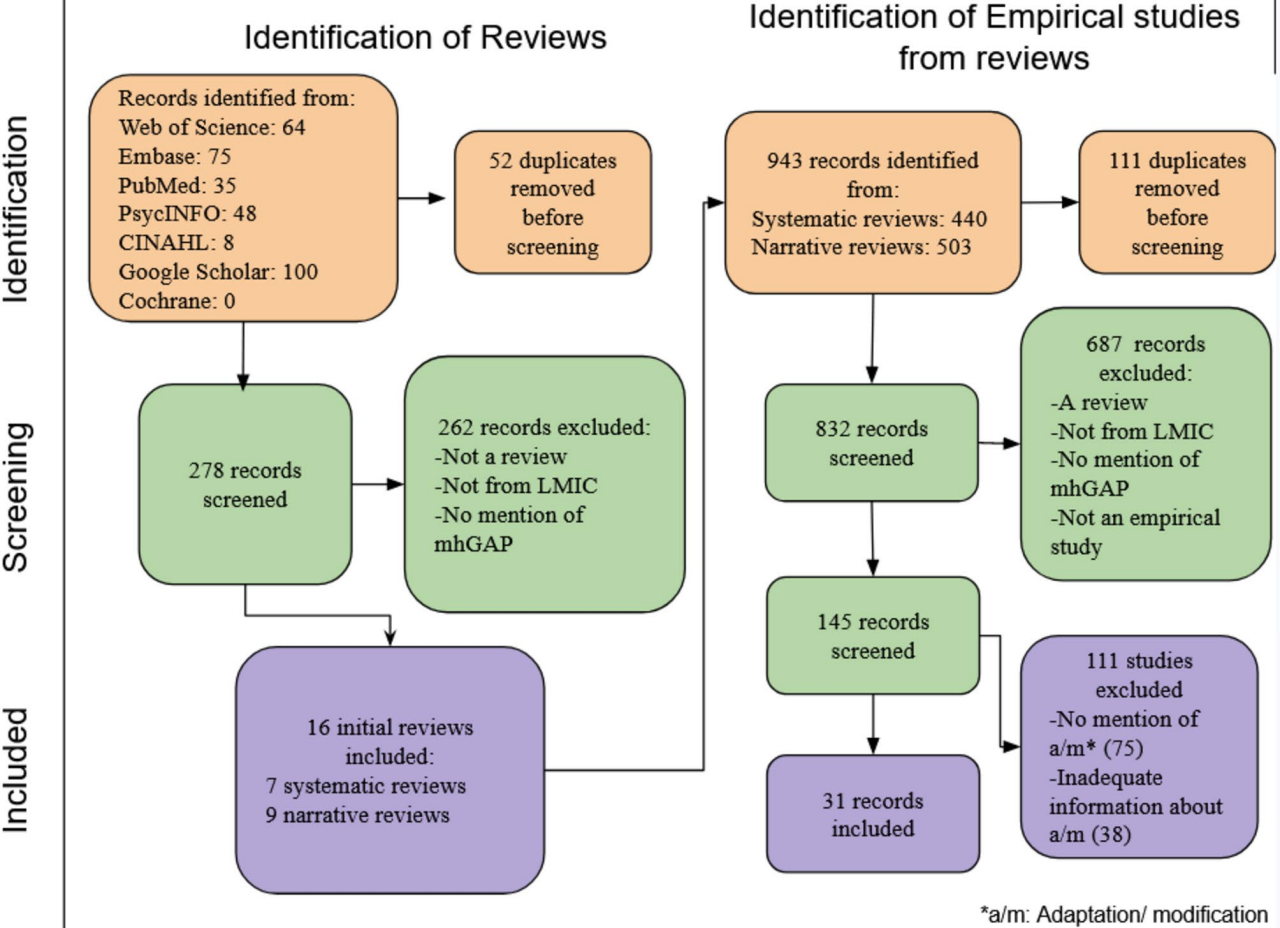
PRISMA Flow Chart

### Screening

At the end of Stage 1 screening, 16 reviews were retained, 7 of which were systematic reviews and 9, narrative reviews or commentaries (Fig. 1). 943 studies were extracted from these 16 reviews (Table 2). At the end of Stage 2 screening, 156 studies were retained and included for analysis. During data extraction, an additional 11 studies were excluded. Of the 145 studies remaining, 69 mentioned modifications or adaptations and 76 did not. Out of the 69 studies that mentioned modifications or adaptations, 38 studies merely mentioned adaptations but without descriptions where information could be extracted or cited versions of adaptations that already available in their country or cultural context. The rest (*n* = 31) mentioned and described adaptations or modifications in sufficient detail for data extraction and analysis and were thus included.

**Table 2:** Summary of the reviews screened in stage 1 and studies extracted at beginning of stage 2

Lead Author and Year	Type of Review	Title	Studies Extracted
Cénat 2020 [28]	Systematic	A systematic review of mental health programs among populations affected by the Ebola virus disease	11
Raj 2021 [29]	Systematic	Child and Adolescent Mental Health Training Programs for Non-specialist Mental Health Professionals in Low and Middle Income Countries: A Scoping Review of Literature	7
Spagnolo 2021 [30]	Systematic	Implementation and use of the Mental Health Gap Action Programme Intervention Guide (mhGAP-IG): A review of the grey literature	151
Keynejad 2018 [10]	Systematic	WHO Mental Health Gap Action Programme (mhGAP) Intervention Guide: a systematic review of evidence from low and middle income countries	33
Keynejad 2021 [11]	Systematic	WHO mental health gap action programme (mhGAP) intervention guide: updated systematic review on evidence and impact	162
Babatunde 2021 [31]	Systematic	Barriers and facilitators to child and adolescent mental health services in low-and-middle-income countries: A scoping review.	28
Koly 2021 [32]	Systematic	Educational and Training Interventions Aimed at Healthcare Workers in the Detection and Management of People With Mental Health Conditions in South and South-East Asia: A Systematic Review.	48
Mills 2019 [33]	Narrative	‘Built for expansion’: the ‘social life’ of the WHO’s mental health GAP Intervention Guide	72
Faregh 2019 [6]	Narrative	Considering culture, context and community in mhGAP implementation and training: challenges and recommendations from the field	95
Dua 2016 [34]	Narrative	Discussion of the updated WHO recommendations for mental, neurological, and substance use disorders	11
Gómez-Carrillo 2020 [35]	Narrative	Engaging culture and context in mhGAP implementation: fostering reflexive deliberation in practice	73
Iversen 2021 [36]	Narrative	Enhancing mental health pre-service training with the WHO mhGAP Intervention Guide: experiences learned and the way forward	11
Katchanov 2012 [37]	Narrative	Epilepsy care guidelines for low- and middle- income countries: From WHO mental health GAP to national programs	33
Ventevogel 2014 [38]	Narrative	Integration of mental health into primary healthcare in low-income countries: Avoiding medicalization.	130
Hughes 2019 [39]	Narrative	mhGAP– the global scenario	22
Uwakwe 2014 [40]	Narrative	Public Mental Health– Using the Mental Health Gap Action Program to Put all Hands to the Pumps	56
		**TOTAL**	**943**

### Study characteristics

Most of the studies employed mixed methods (*n* = 20), followed by quantitative (*n* = 7) and qualitative (*n* = 4) methods alone. All included studies were conducted in the primary care setting and/or community-based settings.

#### Geographical distribution of studies

**Fig. 2:**
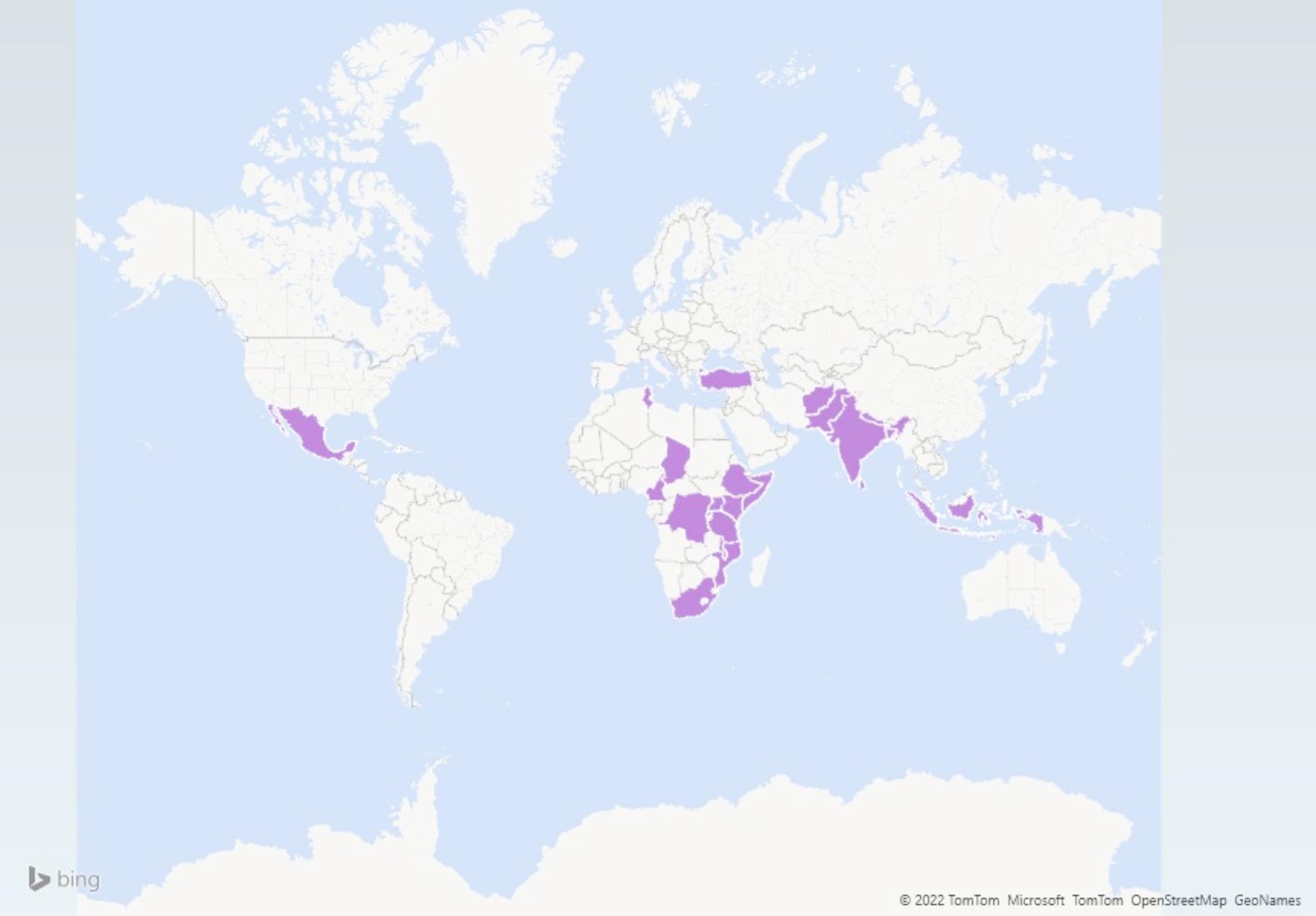
A map highlighting the countries where the final selected studies were conducted

All 31 studies were published between the years 2015 and 2020 and represent 25 countries covering five of the six WHO regions (Fig. 2). Most of the included studies were from the African Region (*n* = 15), and the South-East Asian Region (*n* = 11), with some studies from the Eastern Mediterranean Region (*n* = 1), Region of the Americas (*n* = 2), and European region (*n* = 1), as well as grouped multiple regions (*n* = 2). No studies identified were from the Western Pacific Region. One study was simultaneously conducted in India, Nepal, Uganda, Ethiopia, and South Africa (PRIME) [5] and one was implemented in 6 countries including Ethiopia, India, Nepal, Nigeria, South Africa, and Uganda (EMRALD) [40].

## Analysis as per FRAME

### (i) when and how in the implementation process the modification was made? (ii) whether the modification was planned/proactive or unplanned/reactive

Studies highlighted that most of the modifications were carried out prior to the implementation of the interventions. All modifications were made in a proactive approach except one where modification was made after a pilot initiative [41]. In this exception, Grelloti and colleagues (2015) implemented a pilot prior to adaptation and full scale implementation of the adapted version of mhGAP-IG guidelines to augment the community mental health services in the aftermath of the 2010 earthquake in Haiti [41].

### (iii) who determined the modification?

The decisions to modify mhGAP were necessitated and effectuated by researchers [40–59], research and educational institutes [60], government ministries and departments [40, 42, 44, 46, 49–51, 58, 60–68], programme leaders [43, 69], health care professionals [41, 64, 66, 67] including mental healthcare professionals [57], the World Health Organisation [62, 64], decentralised government bodies and planning units [70], non-governmental organisations [66, 70], armed forces [54] and team of experts who developed similar programme [56, 69].

During the systematic review, we were also able to identify the stakeholders who were involved in the process and were actively involved in adapting or altering the intervention. A wide array of stakeholders were part of the process of decision making including primary health care service providers and supervisors (including non-specialist healthcare workers) [41, 42, 48, 52, 60, 61, 67], mental health care professionals (psychiatrists and psychologists) [41–43, 48, 52, 56, 60–62], policy makers [40], researchers [54, 56], neurologists [42, 62], government officials [40, 42, 45, 46, 49–51, 58, 63] and representatives from NGOs [45]WHO collaborating centres [49–51], staff and consultants [67]; health service organisations [68] and research and educational institutions ^37, 46–48, 54^. In some studies, linguists [48, 52], translators [41], dramatists [56], sociologists [48, 52] were also included in the process. Furthermore, some studies also involved members of the community to include community leaders, volunteers, traditional and religious leaders [45, 68]. Voices of patients and their caregivers were included too [45, 46, 68]. In most of the studies multiple stakeholders were a part of the decision-making process for implementing adaptations.

### (iv) WHAT is modified?

#### Content modification

Several studies encompassed a diverse spectrum of MNS disorders, as outlined as modules in the mhGAP-IG [44, 48, 52, 54, 56, 64, 65, 67, 68]. Some studies embraced a more targeted approach by incorporating specific disorder modules like depression, substance abuse, etc. guided by local needs assessments or aligned with national agendas [40, 43, 45–47, 49–51, 55, 57, 59, 66]. Additionally, certain studies addressed the needs of vulnerable populations, such as refugees and those affected by events like earthquakes [53, 61, 69]. Other studies did not specify any specific disorders [41, 42, 58, 60, 63, 70]. Certain modules in mhGAP-IG were prioritised based on national priorities and local contexts [40, 43, 45, 46, 54, 59, 60, 62, 64, 65, 67, 68]. For example, Dos Santos and colleagues (2019) selectively focussed on epilepsy owing to governmental initiatives and priorities focussed at reducing epilepsy treatment gap. In contrast, Humayun and colleagues (2017) incorporated stress-related and dissociative disorders, which were more prevalent among the internally displaced population in Pakistan.

Further, content within modules were modified by means of simplification of language [42], translation for delivery in local languages [41, 44, 46–48, 51, 52, 55–57, 62, 64, 70] and creation of videos in local languages [43, 55, 56]. Local terms for disorders, their manifestations and common management practices were included [41, 60, 64]. Local elements pertaining to the disorders were brought in, for example, local means of attempting suicides such as ingestion of pesticide (given their availability and affordability depending on location and setting), familiarisation with local names of substances, etc [49, 51]. Additional content not part of the mhGAP guidelines were also added [41, 54, 64, 67]. For example, mental health promotion was included as part of mhGAP training modules [67] and inclusion of anxiety disorders and PTSD [41].

Prescription of drugs and treatment/ management approaches (based on availability, accessibility in country) and indications as to when a referral was warranted were specified [57]. In addition, the challenges pertaining to the accessibility of pharmaceuticals were addressed through the incorporation of locally available essential medications into the training curriculum, alongside the delineation of specific clinical scenarios in which they should be prescribed as part of the training [51].

Momotaz and colleagues (2019), highlighted a clear division of roles and responsibilities of different health care providers in the intervention program by placing a notable emphasis on psychosocial interventions within each training module with health care workers and agencies, while explicitly stating the responsibility for pharmacological management rested with physicians’ training [64]. Due to resource constraints and the inherent vulnerability of the target population, Humayun and colleagues (2017) decided to shift the primary focus of the intervention towards psychosocial management of substance abuse and omitting the need for drug replacement therapy [54].

Furthermore, innovative methods were used for cultural adaptation of the psychoeducational content outlined in the mhGAP-IG such as the use of local artists to draw relevant sketches and inclusion of pictorial representation and spread awareness about mental health concerns among community members and cater to the needs of those with low literacy [69].

#### Contextual modification

Contextual modifications mostly were related to format of delivery. The medium of delivery was made virtual to improve reach and ensure better standards, and consistency. Musyimi and colleagues (2016) designed a mobile phone application of the mhGAP-IG depression component [47], Khoja and colleagues (2016) included mhGAP as a feature for screening and management of MNS disorders on a mobile based experience [58] and Maulik and colleagues (2016) delivered the intervention via a mobile based electronic decision support system [57].

Information, education and communication materials to accompany the mhGAP-IG and training were prepared in local languages in an attempt to increase awareness and reduce stigma [62]. Locally accepted and prevalent theories of disease causation and belief systems associated with them were also taken into consideration in an attempt to foster help-seeking behaviour and minimise stigma in the community to ensure greater uptake of treatment in the long term [70]. Patel and colleagues (2017) included home-based delivery, use of pictorial patient resources, strategies to encourage and include significant others in the treatment process and peer supervision to ensure sustainability and enhance efficacy of implemented mhGAP interventions [59]. Miguel Esponada and colleagues (2020) also included individual and group talk-based interventions and home visits to normalise mental health-related conversations [66]. Extended family was involved in sessions to improve the efficacy of the intervention in the context of pregnant women [69]. Promotion of mental health was added as an important module in the adapted version of the intervention [67]. Peer support groups were created for the recipients and caregivers in an attempt to facilitate psychosocial support among the community members [46].

#### Training, evaluation, implementation and scale-up

In response to the challenge posed by the heterogeneous composition of healthcare professionals within the primary care setting, segregation of participants into two distinct groups: clinicians and community-based workers, was done in an endeavour to impart an appropriate level of knowledge and skill set tailored to the unique roles and backgrounds of these distinct categories [44]. The content was augmented with role plays, group discussions, examples, case illustrations and vignettes relevant to the local settings [43, 50, 51, 55–57, 61, 68]. Implementation and scale-up were catered for by ensuring continuity of training. Tutors were included in the intervention to assist the trainees during and after the training [49]. Refresher trainings were also included [64]. Cost analysis tools were employed, and forward-looking projections were generated to assess the feasibility and devise strategies for the sustainable implementation and potential scalability of the adapted interventions [40, 68].

### (v) at what LEVEL OF DELIVERY the modification is made

Most of the modifications in mhGAP were made at the population level, i.e., the modifications were intended for application to particular cultural contexts [42–44, 46–51, 53–65, 70]. Some of these interventions were modified at the cohort level, i.e., keeping in mind the needs of targeted groups such as children and adolescents [61], refugees [44, 67], maternal group [53]Pregnant women with mental health problems [69], those suffering from depression [47, 59] and post conflict population (internally displaced people and returnees) [65].

### (vi) type or nature of content-level modifications

In the context of the systematic review, nature of modification encompassed tailoring (minor changes to the intervention leaving all major intervention principles and techniques intact) [41, 42, 44–46, 48, 49, 52–57, 61–64, 66–70]; adding (additional materials or activities inserted that are consistent with fundamentals of interventions) [49–51, 61, 67] and removing (not including particular elements of interventions) [40, 42, 45, 46, 53, 55, 60, 62, 67, 69] elements from the mhGAP modules. Some studies also involved condensing [43, 59, 61, 65] of the content of the modules, i.e. time allocated to the prescribed interventions’ content. Sibeko and colleagues (2018) integrated parts of the mhGAP with other frameworks and guidelines, namely, South African National framework and UNESCO guidelines in an attempt to develop a holistic intervention for community health workers training [70].

### (viii) what was the GOAL?

Within the conceptualisation of the FRAME, the modifications were aimed at increasing reach and engagement [40–42, 44–46, 49–51, 53–57, 59, 62, 64–66, 68–70], addressing cultural factors [41, 43, 44, 46, 49–51, 53, 55, 56, 59, 63, 64, 66, 67, 70], improving fit with recipients [55, 59, 65–67, 70], increasing satisfaction [44], improving feasibility [47, 48, 52, 61] and effectiveness [46] and reducing cost [40, 45, 46, 56, 64, 68].

In the studies, mhGAP was mostly modified owing to sociopolitical [40–52, 56–58, 60–64, 66–68, 70] and organisational reasons [40–52, 57, 58, 60–65, 68–70]. The sociopolitical reasons included the nature of existing laws, policies and regulations, availability of resources/ funding and/or evolving societal/ cultural norms. Organisational reasons included availability of resources, such as funding, staff, space, increased accessibility, leadership support, and service structure. Recipient related reasons for modifications included a lack of knowledge, confidence and attitudes [54, 55, 64, 70] and vulnerability of target intervention groups [53, 59, 65, 69]. In addition, some studies mentioned provider related reasons for modification of mhGAP such as clinical judgement, preferences, perception of interventions, previous training, skills and competencies [53–55, 57, 59, 64, 68, 69]. It is pertinent to note here that the majority of the studies reported multiple reasons for modification.

### Additional analysis

#### Process of formulating adaptations or modifications to interventions

Some studies mentioned the processes followed to modify. These included a formal situational analysis [42, 45, 60, 68], asset mapping [40, 45], prioritisation exercise [40, 68], comprehensive review of literature [42, 45], review of existing curriculum [61], stakeholder involvement by means of consultative workshops, as well as interviews and focus group discussions [40, 42, 45, 60, 68]. These processes were often followed by the pilot testing of modules [60, 68] prior to large scale implementation. Some studies undertook costing exercises as well to gauge the fiscal feasibility of the modification processes [40, 68]. An additional component of the process of modifications was seeking feedback from third parties (experts not part of developing the adaptation), providers (managers and co-ordinators), recipients or users (patients, caregivers) of the intervention [44].

## Discussion

Modifications are important to the successful delivery of interventions in diverse settings. They improve upon the original intervention, which is often universally designed, by capturing different local contextual factors. The study aimed to characterize modifications to mhGAP-IG for implementation in LMICs using FRAME [16], understand the nature and processes involved in modifications, and propose any relevant modifications to the framework. To our knowledge this is the only such review to specifically characterise modifications of mental health care treatment packages for delivery in LMICs. By better understanding the types of modifications that have been used, and across different settings, this can ultimately inform efforts to enhance the scalability and impact of interventions aiming to implement solutions to improve access to mental health care.

Almost all the modifications we uncovered were pre-implementation and planned, whereas no modification was unplanned or reactive. This could be attributed to the inherent requirements for adapting mhGAP modules to the local context outlined in the guidelines [4]. This may also be because reactive modifications may be often unnoticed and hence poorly documented, or not documented at all. This needs to be explored.

Although the goal of modification is to respond to unique community contexts, the review finds that the process of modification is often led from the top, that too by researchers, with local stakeholders including the ministries of health only participating in a consultative role. This may be reflective of the nature of funding in global mental health which often drives the mental health delivery in individual countries. Because of this nature of who decides to modify, it is unsurprising that almost all the modifications made were proactive and planned, which is also in line with the recommendations of the mhGAP itself.

Despite this top-down approach, the modification processes are quite consultative, participatory and make use of innovative methods that build on local guidelines, local evidence and local expertise. The involvement of multiple stakeholders helps establish a common understanding of the issues at hand, foster trust among those participating, promotes long-term capacity development by creating pathways for coordinated action [71], thereby enhancing the potential acceptance and adoption of interventions within the community. Most of the modifications were to the content, the delivery, training of mhGAP and there were minimal reports on modifications in scale-up which may refer to the early stages of global mental health delivery at the primary care level.

The studies included in the review highlight the need to adopt an emic approach to understanding and improving mental health interventions. This includes the need for exploring the perspectives, explanations, logic, meanings, beliefs and worldview of people so as to explain their values, beliefs or practices [72]. Adapting mental health interventions to the cultural contexts of specific communities and individuals will help improve the relevance, efficacy, acceptability and sustainability of the interventions; subsequently contributing to significantly reducing treatment gaps and disparities in delivery and uptake of mental health care [73]. In LMICs, where resources are limited, modifying interventions to local needs can help reduce treatment gaps and improve access to care. Modifications should be iterative, wherein emerging data, global practices, changing local resources and changing awareness and knowledge about cultural perceptions and determinants of mental health in local populations in the course of implementing modifications should continually inform and enhance the refinement, customization, and execution of adapted interventions [64, 74–76].

Despite significant progress in the science of modifications and adaptations, further progress may be attenuated by the limited reporting of modifications. This includes not reporting on challenges, process of modification, outcomes, timely audits and evaluations, lack of use of modification frameworks, critical analysis and appraisals of the implementations. Since design and implementation of modifications de novo require significant financial and human resources, time and expertise, adequate reporting of modifications is important as modifications in similar cultural contexts can act as an important guide that can facilitate better planning and execution, ensure adequate allocation of resources, and prevent duplication of efforts.

### Use of FRAME for studying modifications

We find FRAME as a valuable tool to document modifications. By covering a wide range of aspects of modifications FRAME provides a comprehensive overview of modifications. Yet, based on our findings, we suggest that FRAME could be further enhanced by these modifications to the framework:

Within existing elements in the framework, in addition to the list of participants suggested we found that other participants include external organisations such as WHO, other countries, and armed forces. Similarly, in terms of the suggested list of goals in the FRAME, some studies report other goals such as capacity building (infrastructural and human resources) and making the adaptation lucrative for policy makers.

Outside of existing elements, new elements may be added to the FRAME, especially with respect to documenting the process of modification.

I. *Sources of knowledge*: Several studies detail the source of knowledge used for designing the modification. Examples include: The WHO Assessment Instrument for Mental Health Systems (WHO-AIMS) [42, 60], International Association of Child and Adolescent Psychiatry and Allied Professionals textbook^59^, WHO-SPE STRESS Intervention Guidelines [54], the book “Where there is no psychiatrist” [55], UNESCO Training Guide and Best Practice Guidelines for Implementing and Evaluating Child and Adolescent Well-being Programs in Healthcare Settings [70]. In addition to these other sources, specific methods were used for planning the modifications such as focus group discussions, key informant or expert interviews. Some studies report use of existing modifications that have been reported in other studies [42].
II. *Financial and temporal aspects*: Furthermore, the FRAME does not provide any information pertaining to the financial and temporal aspects related to the implementation of interventions [77, 78]. In addition, information on the human resources involved in the modification would also be useful information [70]. An estimation of the time and resources required can help interventionists better allocate resources and improve efficiency.

### Modifications and their relationship to fidelity

The FRAME classifies modifications as fidelity-consistent (preserving core elements) and fidelity-inconsistent (fail to preserve its core elements). The relationship to fidelity can be made in consultation with existing literature, inputs from the developer and evaluation data for the implementation programme. In the context of the current study, however, delineating this difference was challenging as it may be noted that core and non-core elements of mhGAP-IG have not been outlined. Moreover, most of the reports on modifications do not sufficiently report whether modifications were fidelity consistent or inconsistent. This is in line with other systematic reviews [79]. Hence, it is recommended that future studies report core and non- core elements of interventions.

### Limitations

Only studies reviewed in previous studies have been included. Grey literature was not included in this systematic review, although earlier reviews included in Stage 1, such as by Spagnolo et al. (2021), reviewed grey literature. This may mean that less significant information on modifications, which may have been reported only in grey literature, was missed. No numerical assessments of bias were done and the data were too heterogeneous for meta-analysis. While disagreements between independent reviewers during screening and data extraction were discussed and resolved by iterative consultations until consensus was reached, the process was not followed for other attributes and aspects of the study as data synthesis and analysis were done primarily by HR and AR with supervision of JN and JS. For example, this included decisions on whether a modification characteristic fell under the *content* or *context* element of FRAME. We also did not reach out to other authors of studies that did not adequately describe the modifications/ adaptations for additional unreported data.

## Conclusion

Modifications are crucial for making interventions responsive to diverse community settings. But this review shows that they are often led by researchers in a top-down manner, with local stakeholders in a consultative role. Despite this, the process remains participatory, drawing on local guidelines and expertise. FRAME is a valuable framework for documenting modifications and can be improved to capture key aspects like sources of knowledge, financial and temporal aspects. However, limited reporting on modifications in implementation science literature, especially with regards to challenges and outcomes, hinders progress. Better documentation can guide future modifications, optimize resource allocation, and enhance implementation and scale-up outcomes in similar cultural contexts.

## Data Availability

No datasets were generated or analysed during the current study.
